# Multiple cutaneous and intestinal metastases in lung cancer: A case report

**DOI:** 10.3892/ol.2015.2893

**Published:** 2015-01-26

**Authors:** SHOUTANG LU, JIANSHU YANG, YANLAI SUN, ZHONGFA XU

**Affiliations:** 1 Department of Gastrointestinal Surgery, Affiliated Hospital of Shandong Academy of Medical Sciences, Jinan, Shandong 250031, P.R. China; 2 Department of Colorectal Cancer Surgery, Shandong Cancer Hospital, Jinan, Shandong 250117, P.R. China; 3 Shandong Academy of Medical Sciences, Jinan, Shandong 250062, P.R. China

**Keywords:** lung cancer, cutaneous metastasis, intestinal metastasis, pathological biopsy

## Abstract

Lung cancer is a common malignant neoplasm that is prone to distant metastasis. However, the incidence of multiple cutaneous and intestinal metastases is rare. The present study describes the case of a 62-year-old female who was admitted to The Affiliated Hospital of Shandong Academy of Medical Sciences in August 2013 with multiple cutaneous lumps. Contrast-enhanced computed tomography showed nodules and masses in the right lung, and multiple enlarged lymph nodes in the mediastinum and right hilum. Biopsies of the lumps in the right lung and skin revealed moderately-differentiated adenocarcinoma, which were considered to be cutaneous metastases of lung cancer. The patient subsequently experienced symptoms of rectal irritation. A digital rectal examination and colonoscopy were performed, and the consequent pathological biopsy identified moderately-differentiated adenocarcinoma. After analyzing the results of previous pathological examinations and immunohistochemistry, it may be suggested that intestinal metastasis had developed. This case highlights the fact that a comprehensive analysis and examination should be performed for suspected cutaneous and intestinal lesions, during which, a pathological biopsy is of great importance in order to form the correct diagnosis for timely treatment.

## Introduction

Lung cancer is one of the most common tumors globally; it is highly malignant and has a high rate of distant metastasis (1). In total, ~50% of these patients already have distant metastasis when diagnosed, with the most common metastasis sites being the lungs, liver, bone, brain and adrenal glands (1). Cutaneous metastasis of lung cancer is rare, its pathogenesis is by either lymphovascular invasion or hematogenous metastasis (2). The histology of cutaneous metastsis most commonly reveals adenocarcinoma, then squamous/small-cell carcinoma, followed by large-cell carcinoma (3). Common treatment modalities include surgery, chemotherapy and radiotherapy. Currently, the prognosis for patients with cutaneous metastasis of lung cancer is poor. Intestinal metastases from lung cancer are rare and the diagnosis is often late, with clinical symptoms of bowel occlusion and intestinal bleeding (4). In certain cases the clinical manifestations of the metastases have been observed prior to those of the primitive tumour (5). However, in the presence of bowel occlusion and intestinal bleeding of uncertain origin, obtaining a clinical history is particularly important and diagnostic procedures must be performed to rule out a secondary pathology (6). Until now, simultaneous cutaneous and intestinal metastases have never been reported. The present study reports such a case that was recently admitted to The Affiliated Hospital of Shandong Academy of Medical Sciences (Jinan, China). Written informed consent was obtained from the patient.

## Case report

**Figure 1:**
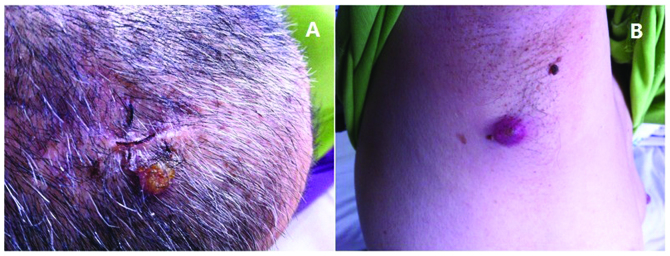
Clinical photograph showing (A) a lump on the top of the head and (B) a purple, protruding lump in the right armpit.

**Figure 2:**
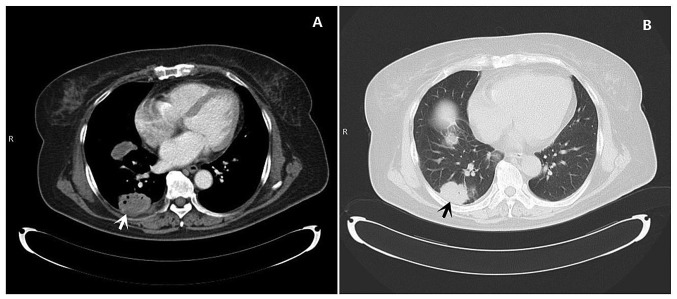
Chest computed tomography showing the primary tumor in the inferior lobe of the right lung (arrow) in the (A) mediastinal and (B) pulmonary windows.

**Figure 3:**
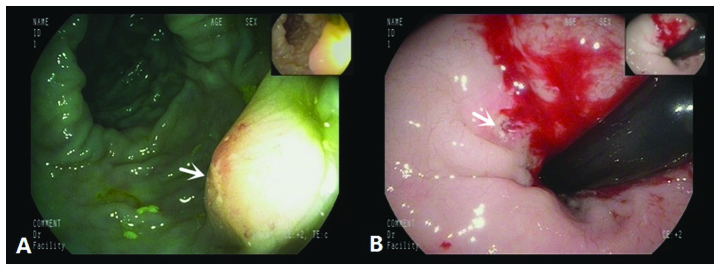
Electronic colonoscopy images showing (A) a 0.3×0.3-cm lump in the transverse colon that exhibited a rough mucosal membrane on the top, with clear boundaries and (B) an ulcer with a diameter of ~1 cm, a recessed center, a peripheral bulge and a hard texture on the rectum.

**Figure 4:**
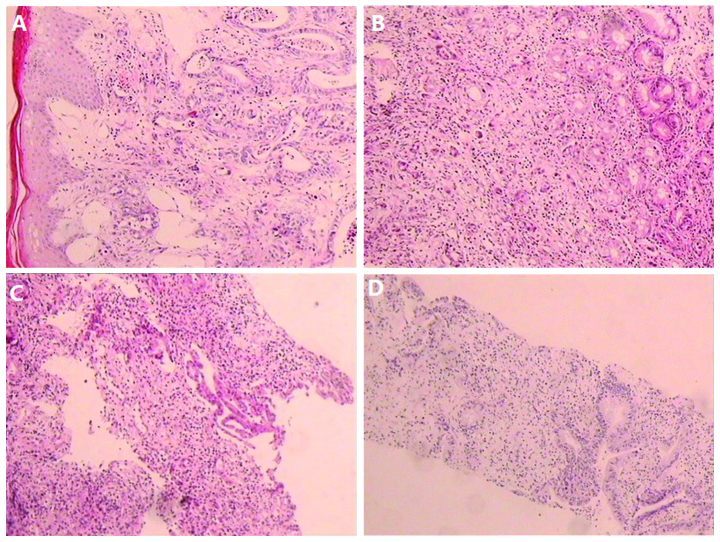
Histopathology of (A) cutaneous metastasis, (B) rectal metastasis, (C) transverse colon metastasis and (D) the primary tumor of the right lung. All were histopathologically diagnosed as moderately-differentiated adenocarcinoma (hematoxylin and eosin; original magnification, ×100).

A 62-year-old female was admitted to The Affiliated Hospital of Shandong Academy of Medical Sciences in August 2013 with multiple lumps in the right thigh, armpit and scalp that had been present for one month. A number of these lumps had ulcerated two weeks prior to the visit. Three lumps were observed on the scalp, among which the top lump was the largest. This lump was a hard, 3×2 cm protrusion, which was recessed and ulcerated at the center, with a clear embankment-like boundary. The other two bulges looked like craters, with clear boundaries and no ulceration or exudation. No tenderness was reported. In addition, a furuncle-like lump was found on the right thigh, which was swollen and ulcerated, with mild tenderness. A purple, protruding 2×2-cm lump could also be observed in the right armpit, with furuncle-like embossing of the top and clear boundaries. The lump was of moderate texture, with a certain degree of tenderness (Fig. 1). Upon physical examination, chest auscultation revealed clear breathing sounds for the left lung, while those of the right lung were comparatively lower. There was no rhonchus or moist rale and other parameters were normal. Chest computed tomography (CT) showed nodules and masses in the right lung, with multiple enlarged lymph nodes in the mediastinum and right hilum (Fig. 2). This suggested a diagnosis of primary right lung cancer with intrapulmonary and lymphatic metastases in the mediastinum and right hilum. Resection of the tumors on the scalp, right thigh and armpit was performed due to the ulcerated cutaneous nature of the tumors. Pathological examination showed moderately-differentiated adenocarcinoma, which was considered to be metastatic cancer. A percutaneous biopsy of the right lung tumor showed moderately-differentiated adenocarcinoma. During the hospitalization period, the patient experienced increased stool frequency without obvious cause, which included tenesmus with blood and pus, but no abdominal pain, nausea or vomiting. A digital rectal examination revealed blood and a 4×3-cm lump at the rear of the perineal area, which was compressing the rectum. Colonoscopy showed a 0.3×0.3-cm lump on the inside of the transverse colon, which exhibited a rough mucosal membrane on the top, with clear boundaries. An ulcer with a diameter of ~1 cm, a recessed center, a peripheral bulge and a hard texture was observed on the rectum (Fig. 3). Biopsies were taken from the two sites, which were both subsequently diagnosed as moderately-differentiated adenocarcinoma. A similar pathology as that shown on light microscopy (Fig. 4) and similar immunohistochemistry results (Table I) indicated that the tumors in the intestines, scalp and thigh were all metastases of the primary lung cancer. The patient is currently undergoing systemic chemotherapy with intravenous gemcitabine (1.4 g, days 1 and 8) and cisplatin (40 mg, days 1–3) every two weeks. At the time of writing, the patient had undergone eight weeks of a six month treatment. However, as the patient exhibits multiple cutaneous metastases and intestinal metastasis, the expected surival time is poor.

## Discussion

More than half of all lung cancers have metastasized when diagnosed. These metastases occur most often in the thoracic lymph nodes (46–85%), pleura (14–46%), brain (14–45%), adrenal gland (36–64%), bone (21–41%), contralateral lung (13–43%) and kidney (13–43%), and a small percentage of metastases are in the abdominal lymph nodes, spleen, pancreas, heart, pericardium and other regions (1,7). Cases of lung cancer with cutaneous and intestinal metastases are rare.

Cutaneous metastasis is caused by primary cancer-derived cells that grow in the skin (8). According to the published literature, the incidence of cutaneous metastasis is 2.9–5.3% in general (9), and 1–12% for lung cancer (10). Clinically, cutaneous metastasis often manifests as single or multiple nodules, possibly at multiple sites (usually close to the primary tumor), which differ in size (often 0.5–10 cm), and are round or oval, hard, relatively immobilized, normally colored, purple or bright red, and ulcerated or cauliflower-like, with bleeding in certain cases (8,11). Certain cases may also manifest as erysipelas-like cancer, vascular dilation or bullous-like lesions, papules, plaques or scarring (12). For the majority of cases, cutaneous metastasis occurs during the progression of primary tumor following the initial diagnosis, but for a few cases, cutaneous metastasis is found prior to the primary tumor or simultaneously with the latter (7,10). Cutaneous metastasis of the breast and oral cancers often result from hematogenous and lymphatic metastasis, where the latter pathway is pivotal, while for other tumors, hematogenous metastasis is usually the primary cause. Metastasis through the lymphatic pathway may explain why cutaneous metastasis occurs proximal to the primary tumor (13). Lung cancer-derived cutaneous metastasis cannot be differentiated from cutaneous metastases of other sources based on the gross specimen. Lung cancer-derived cutaneous metastases are most commonly from lung adenocarcinoma, followed by squamous cell carcinoma, small cell lung cancer and large cell lung cancer (14). Lung adenocarcinoma is usually derived from bronchial epithelial goblet cells, usually the borderline type, and is often asymptomatic in the early stages and not diagnosed till the later stages when metastasis or compression symptoms occur. The prognosis of cutaneous metastasis of lung cancer is poor, and despite the use of chemoradiotherapy, the median survival time is only 3–6 months (11).

Intestinal metastasis of lung cancer is even rarer than cutaneous metastasis, with an incidence of ~0.19% according to the published literature (15). Intestinal metastasis causes abdominal symptoms such as abdominal pain, bloating, bowel dysfunction and bleeding. CT or even positron emission tomography-CT examinations rarely detect small metastases, and false-negative results are common. Patients may initially present with intestinal symptoms rather than typical signs of primary lung cancer due to a lack of specific symptoms. The intestinal metastasis diagnosed by colonoscopy may be misdiagnosed as the primary tumor, therefore a biopsy is required for an accurate diagnosis (16). The path of the intestinal metastasis of lung cancer is currently unclear, although it is generally considered to be lymphatic or hematogenous. However, from previous clinical experience we propose the following two possibilities: i) Metastasis may have occurred through the paravertebral venous system to the intestinal mucosa; or ii) since the patient had a long-term cough with sputum, the cancer cells may have be coughed up with the sputum and swallowed into the digestive tract, where they adhered to the intestines and became established as metastasis. As the patient in the current report exhibited no obvious cough, intestinal metastasis was potentially the result of lymphatic and hematogenous metastaiss. There is currently no solid evidence to support these hypotheses, so relevant basic research is required. Intestinal metastasis indicates an advanced grade of lung cancer, which leaves palliative treatment and supportive care as the only treatment options (17). The median survival time is only 4–8 weeks after the diagnosis of intestinal metastasis of lung cancer (18).

The patient in the present study exhibited no obvious cough or any abnormal lung-related signs or symptoms, and originally presented with multiple cutaneous lumps whose characteristics were similar to that reported by the literature. The primary cancer was identified during the CT examination, and intestinal metastasis was detected by digital rectal examination, colonoscopy and biopsy due to rectal irritation. In summary, the patient exhibited multiple cutaneous and intestinal metastases, possibly the additive result of lymphatic and hematogenous metastasis. The diagnosis of this case was quick, and the patient is currently undergoing systemic chemotherapy with gemcitabine plus cisplatin. As the patient already exhibits multiple cutaneous metastases and intestinal metastasis, the expected survival time may not be long. The efficacy and prognosis of the treatment requires further observation and analysis.

In conclusion, lung cancer is highly malignant and prone to distant metastases, however, cutaneous and intestinal metastases are rare. The present patient originally presented with cutaneous metastases, and the primary tumor and intestinal metastasis was only found during the examination, after which systemic chemotherapy was administered. For suspected cutaneous and intestinal lesions, a comprehensive analysis and examination should be performed, including a timely pathological biopsy according to the characteristics of the cutaneous lesion and a digital rectal examination. A colonoscopy plus biopsy should be routinely used for intestinal lesions to obtain an accurate diagnosis, so that the correct treatment can be applied quickly and patient survival can be prolonged.

## Figures and Tables

**Table I:** Immunohistochemistry results of primary tumors and corresponding metastases.

Primary and metastatic sites	Immunohistochemistry result
Primary lung cancer	CK7(+); CDX2 spotty(+); CK20(−); TTF-1(−); vimentin(−)
Cutaneous metastasis	CK7(+); CK19(+); CA19-9 spotty(+); CDX2 scattered(+); CK20(−); TTF-1(−); GCDFP-15(−); ER(−); PR(−)
Rectal metastasis	CK7(+); CK19(+); CDX2 spotty(+); CK20(−); TTF-1(−)
Transverse colon metastasis	CK7(+); CK19(+); CDX2 spotty(+); CK20(−); TTF-1(−); villin(−)

CK, cytokeratin; CDX2, caudal-type homeobox 2; TTF-1, thyroid transcription factor 1; CA19-9, cancer antigen 19-9; GCDFP-15, gross cystic disease fluid protein 15; ER, estrogen receptor; PR, progesterone receptor.

## References

[1] Jemal A, Bray F, Center MM (2011). Global cancer statistics. CA Cancer J Clin.

[2] Alkhayat H, Hong CH (2006). Cutaneous metastases from non-small cell lung cancer. J Cutan Med Surg.

[3] Song Z, Lin B, Shao L, Zhang Y (2012). Cutaneous metastasis as a initial presentation in advanced non-small cell lung cancer and its poor survival prognosis. J Cancer Res Clin Oncol.

[4] Nishizawa Y, Kobayashi A, Saito N (2012). Surgical management of small bowel metastases from primary carcinoma of the lung. Surg Today.

[5] Berger A, Cellier C, Daniel C (1999). Small bowel metastases from primary carcinoma of the lung: clinical findings and outcome. Am J Gastroenterol.

[6] Cipollone G, Santarelli G, Quitadamo S (2004). Small bowel metastases from lung cancer. Chir Ital.

[7] Jemal A, Center MM, DeSantis C, Ward EM (2010). Global patterns of cancer incidence and mortality rates and trends. Cancer Epidemiol Biomarkers Prev.

[8] Riahi RR, Cohen PR (2012). Clinical manifestations of cutaneous metastases. Am J Clin Dermatol.

[9] Krathen RA, Orengo IF, Rosen T (2003). Cutaneous metastasis: a meta-analysis of data. South Med J.

[10] Mollet TW, Garcia CA, Koester G (2009). Skin metastases from lung cancer. Dermatol Online J.

[11] Triller Vadnal K, Triller N, Pozek I (2008). Skin metastases of lung cancer. Acta Dermatovenerol Alp Pannonica Adriat.

[12] Inamadar AC, Palit A, Athanikar SB (2003). Inflammatory cutaneous metastasis as a presenting feature of bronchogenic carcinoma. Indian J Dermatol Venereol Leprol.

[13] Pathak S, Joshi SR, Jaison J, Kendre D (2013). Cutaneous metastasis from carcinoma of lung. Indian Dermatol Online J.

[14] Sha D, Wang C, Wang W (2009). Skin metastasis of lung cancer: A clinical analysis and review of literature. Central China Medical Journal.

[15] Kim MS, Kook EH, Ahn SH (2009). Gastrointestinal metastasis of lung cancer with special emphasis on a long-term survivor after operation. J Cancer Res Clin Oncol.

[16] Yang CJ, Hwang JJ, Kang WY (2006). Gastro-intestinal metastasis of primary lung carcinoma: clinical presentations and outcome. Lung Cancer.

[17] McNeill PM, Wagman LD, Neifeld JP (1987). Small bowel metastases from primary carcinoma of the lung. Cancer.

[18] John AK, Kotru A, Pearson HJ (2002). Colonic metastasis from bronchogenic carcinoma presenting as pancolitis. J Postgrad Med.

